# Comparative Phenotypic and Genomic Features of Staphylococci from Sonication Fluid of Orthopedic Implant-Associated Infections with Poor Outcome

**DOI:** 10.3390/microorganisms10061149

**Published:** 2022-06-02

**Authors:** Ingrid Nayara Marcelino Santos, Mariana Neri Lucas Kurihara, Fernanda Fernandes Santos, Tiago Barcelos Valiatti, Juliana Thalita Paulino da Silva, Antônio Carlos Campos Pignatari, Mauro José Salles

**Affiliations:** 1 Laboratório Especial de Microbiologia (LEMC), Departamento de Medicina, Escola Paulista de Medicina (EPM), Universidade Federal de São Paulo (UNIFESP), São Paulo 04025-010, Brazil; inayarams@gmail.com (I.N.M.S.); mariana.kurihara@hotmail.com (M.N.L.K.); accpignatari@gmail.com (A.C.C.P.); 2 Laboratório Alerta, Disciplina de Infectologia, Departamento de Medicina, Escola Paulista de Medicina (EPM), Universidade Federal de São Paulo (UNIFESP), São Paulo 04039-032, Brazil; fernanda-fs@hotmail.com (F.F.S.); tiago_valiatti@hotmail.com (T.B.V.); paulinojuliana087@gmail.com (J.T.P.d.S.); 3 Faculdade de Ciências Médicas Santa Casa de São Paulo, São Paulo 01224-001, Brazil; 4 Hospital São Paulo, Universidade Federal de São Paulo (UNIFESP), São Paulo 04024-002, Brazil

**Keywords:** *Staphylococcus aureus*, coagulase-negative *Staphylococci*, biofilm, orthopedic infections, sonication, bacterial resistance, whole-genome sequencing

## Abstract

*Staphylococcus* spp. remain the leading biofilm-forming agents causing orthopedic implant-associated infections (OIAI). This is a descriptive study of phenotypic and genomic features identified in clinical isolates of *S. aureus* and coagulase-negative *Staphylococcus* (CoNS) recovered from OIAIs patients that progressed to treatment failure. Ten isolates were identified by matrix-time-of-flight laser-assisted desorption mass spectrometry (MALDI-TOF-MS) and tested for antibiotic susceptibility and biofilm formation. Genotypic characteristics, including, MLST (Multi Locus Sequence Typing), *SCCmec typing*, virulence and resistance genes were assessed by whole-genome sequencing (WGS). All *S. aureus* harbored *mecA*, *blaZ*, and multiple resistance genes for aminoglycosides and quinolones. All MRSA were strong biofilm producers harboring the complete *ica*ADBC and *ica*R operon. Seven CoNS isolates comprising five species (*S. epidermidis*, *S. haemolyticus*, *S. sciuri*, *S. capitis* and *S. lugdunensis*) were analyzed, with *mecA gene* detected in five isolates. *S. haemolitycus* (isolate 95), and *S. lugdunensis* were unable to form biofilm and did not harbor the complete *icaADBCR* operon. High variability of adhesion genes was detected, with *atl*, *ebp*, *ica*ADBC *operon*, and *IS*256 being the most common. In conclusion, MRSA and CoNS isolates carrying genes for biofilm production, and resistance to β-lactam and aminoglycosides are associated with treatment failure in OIAIs.

## 1. Introduction

*Staphylococci* are the leading pathogens of orthopedic implant-associated infections (OIAIs), including those related to biofilm formation. *S. aureus* and *S. epidermidis* are the most commonly isolated species from OIAI reaching rates above 50% [1]. Methicillin-resistant *Staphylococcus aureus* (MRSA) poses a threat to the management OIAI due to its therapeutic limitations. Likewise, the coagulase-negative *Staphylococcus* (CoNS) group, including methicillin-resistant *S. epidermidis* (MRSE) also plays an important role in OIAIs [2]. Other pathogens of this group with clinical importance are emerging, such as *S. capitis*, *S. haemolyticus*, *S. hominis*, *S. warneri*, and *S. lugdunensis* [3,4].

Most OIAIs start with the introduction of pathogens colonizing the skin and mucous membranes during the surgical procedure. According to the “*race to the surface*” theory, soon after an implant is inserted, competition occurs between host tissue cell integration and contaminating bacteria to colonize the biomaterial surface [5,6,7,8]. The understanding of OIAIs pathogenesis caused by *S. aureus* and *S. epidermidis* is progressing based upon substantial pre-clinical, but few clinical studies [9]. It encompasses extensive amounts of specific proteins (AtlA, AtlE, Bap/Bhp) playing an efficient role in pathogen adhesion to biotic and abiotic surfaces (orthopedic implants) and are called MSCRAMMs (*Microbial Surface Components Recognizing Adhesive Matrix Molecules*). This is followed by bacterial adhesion to- and intra-cellular invasion of osteoclasts, osteoblasts, and osteocytes, small colony variants (SCV) formation within the skeletal cells, biofilm formation with the altered metabolic activity of bacteria resulting in SCV and persisters cells, expression of bacterial resistance genes, and consequent tolerance to antibiotics [4,9,10]. In addition, biofilm, which plays an important role in the pathogenesis of IOAIs, is a community of microorganisms encased by a self-produced structural exopolysaccharide matrix while protected from the immune system of the host and antibiotics. Biofilm-growing (sessile) bacteria are recalcitrant to antibiotic treatment due to multiple tolerance mechanisms, including restriction penetration of antibiotics through the biofilm matrix, restricted growth in the inner part of the biofilm associated to lower levels of oxygen tension, the expression of biofilm-specific genes, resistance genes and the presence of small-colony variants and persisters cells [11,12,13,14].

Studies show that in sessile pathogens, the minimum inhibitory concentration (MIC) of antibiotics can be 1000 times higher than the values used to treat infections by their planktonic counterparts [11]. The mechanisms of biofilm production by *Staphylococcus* spp. are relatively well-characterized and are mainly associated with the production of PIA (*extracellular polysaccharide promoting intercellular adhesion*) or PNAG (*polymeric N-acetyl-glucosamine*), which are synthesized by enzymes encoded by the *ica* operon [12]. Four proteins, coded by the *icaA, icaD, icaB and icaC* genes, are transcribed after the activation of the operon, and the joint expression of *icaA* and *icaD* is particularly efficient in polysaccharide production [13]. On the other hand, PIA-independent surface proteins are also associated with biofilm formation, such as Embp (extracellular matrix binding protein), Bap (biofilm-associated protein), and FnbpA and B (fibronectin-binding proteins) [14]. Despite the current knowledge using in vitro and in vivo animal studies, additional research using clinical isolates is needed to assess the genetic characteristics and pathogenicity of *Staphylococci* associated with OIAI [1,2].

One strategy to improve microbiological diagnosis in OIAIs has been the application of an ultrasonic bath to the surgically removed implants, which increases the sensitivity of culture assays [7,15,16,17]. Moreover, it is essential to progress in the investigation of genomic variations, features of virulence, drug resistance, and biofilm genes, and the outcome of OIAI caused by *Staphylococcus* spp. The study objectives include: (1) antibiotic susceptibility analysis; (2) characterization of resistance phenotypes and genotypes; (3) evaluation of biofilm formation; and (4) detection of biofilm and virulence genes employing complete whole-genome sequencing (WGS) in clinical isolates of *S. aureus*, *S. epidermidis*, *S. haemolyticus*, *S. sciuri*, *S. capitis* and *S. lugdunensis* yielded from sonication fluid of OIAIs patients that progressed to treatment failure. 

## 2. Material and Methods

### 2.1. Study Population and Sample Collection

At a tertiary university center specializing in musculoskeletal infections, clinical, microbiological and antibiotic therapy data were collected from 10 patients with a confirmed diagnosis of OIAI who evolved to poor outcome (failure) after undertaking surgical and antibiotic treatment. Orthopedic surgeries included arthroplasties, osteosynthesis (plate, screws, and intramedullary nail) for stabilization of open and closed fractures of long bones, and spinal osteosynthesis for correction of deformities or degenerative spine disease. The clinical and microbiological diagnosis of OIAI was fulfilled according to the definition of prosthetic joint infection (PJI) and fracture-related infection (FRI) published elsewhere [18,19]. Briefly, the microbiological diagnosis of FRI and PJI requires at least two positive tissue cultures out of at least 4 samples of tissue collected aseptically, with the same low virulence microorganisms (coagulase-negative *Staphylococci*) identified phenotypically. Therefore, the identified CoNS is considered pathogenic when the organism is found in at least 2 different culture samples, including sonication fluid cultures [17]. All patients underwent surgical revision with the removal of the orthopedic implants, which were then submitted to the sonication technique for microbial diagnosis. After surgical removal of the implants in the operating room, each device was immediately placed in a sterile polypropylene container, hermetically closed, identified, and sent to the microbiology laboratory of the Institution. This study was reviewed and approved by the local ethics committee (n. 3.622.166, on 4 October 2019).

### 2.2. Sonication and Microbiological Methods

The implants were sonicated according to the technique of Trampuz et al. [20] and modified by Yano et al. [17]. Briefly, the sonication technique consisted of vortexing the sterilized solid polyethylene containers with the implants and 50 to 250 mL (depending upon the device width) of Ringer solution for 30 s using a Vortex-Genie 2 (Scientific Industries, Inc., Bohemia, NY, USA), and then treat it in an ultrasonic bath (BactoSonic; Bandelin GmbH, Berlin, Germany) for 5 min at a frequency of 40 ± 2 kHz and power density of 0.22 ± 0.04 W/cm^2^, followed by another 30 s of vortexing. Subsequently, a centrifugation step at 600× *g* (2500 rpm) for 5 min was carried out, to concentrate the sonication fluid. The supernatant was aspirated, leaving 0.5 mL (100-fold concentration), and aliquots of 0.1 mL of concentrated sonicate fluid (SF) were then plated on blood agar (Probac do Brasil, São Paulo, SP, Brazil) and incubated for 18–24 h at 37 °C. Colonies of isolated microorganisms cultured on plates were quantified (number of colony-forming units [CFU]/mL of sonication fluid). Due to the addition of a sonicate fluid (SF) concentration step, counts above the cut-off point of 50 CFU/mL were considered positive and used for optimal sensitivity and specificity analyses. *Staphylococcus* spp. were identified by colonial morphology, Gram staining features, the catalase test, and the coagulase test (rabbit plasma), and species were confirmed using matrix-assisted laser desorption/ionization time-of-flight mass spectroscopy (MALDI-TOF MS) (Bruker, Billerica, MA, USA).

### 2.3. Species Identification by MALDI-TOF MS

Ten non-duplicated clinically important isolates, phenotypically characterized as *Staphylococcus* spp., were identified at the species level by MALDI-TOF MS. The extraction of total proteins from each isolate was performed according to the manufacturer’s standards (Bruker Daltonics, Billerica, MA, USA). Spectra were obtained in triplicate for each pathogen, using the Microflex LT mass spectrometer (Bruker, Billerica, MA, USA)., and bacterial identification was based on spectra comparison with those present in the MALDI Biotyper 3.3 software database (Bruker, Billerica, MA, USA ). According to this software, the value ≥ 2.3 indicates that genus and species identification is reliable; a value between 2.0 and 2.29 indicates that the identification of the genus is reliable and the identification of the species is probable, and between 1.7 and 1.9 indicates that gender identification is likely. Values lower than 1.69 indicate that the identification is unreliable and must be repeated.

### 2.4. Antibiotic Susceptibility Tests

The antibiotic susceptibility profiles of the ten isolates of *Staphylococcus* spp. were evaluated by the Kirby-Bauer disk diffusion technique and for the evaluation of MICs, microdilution was performed in broth and E-test based on the criteria and recommendations of the Brazilian Committee on Antibiotic Susceptibility Testing-BrCAST and the European Committee on Antibiotic Susceptibility Testing-EUCAST (BrCAST (http://brcast.org.br/ Accessed in February/March 2020)/EUCAST (https://www.eucast.org/ Accessed in February/March 2020)). Quality control was performed with the standard strains *S. aureus* ATCC^™^,25923, *S. aureus* ATCC^™^ 29213 and *Enterococcus faecalis* ATCC^™^ 29212.

#### 2.4.1. Inoculum Preparation

After being seeded on Blood agar for about 18 h to ensure the purity of the samples, with the aid of the 10µL seeding loop, around 3 to 5 colonies isolated from each sample were transferred to tubes containing 3 mL of saline solution at 0.85%. The bacterial suspension was homogenized and the turbidity measured in a digital turbidimeter (Baxter^®^, Sacramento, CA, USA), to obtain a bacterial concentration of around 1.5 × 10^8^ of colony-forming units (CFU)/mL corresponding to 0.5 of the McFarland scale.

#### 2.4.2. Diffusion Disk Test

The antibiotic susceptibility profiles of the ten isolates of *Staphylococcus* spp. were evaluated by the Kirby–Bauer disk diffusion technique. The following antibiotics were tested: cefoxitin (30 µg), tetracycline (30 µg), clindamycin (2 µg), erythromycin (15 µg), norfloxacin (10 µg), gentamicin (10 µg), rifampicin (5 µg), linezolid (30 µg), chloramphenicol (30 µg) and trimethoprim/sulfamethoxazole (1.25/23.75 µg). All discs were from Oxoid (Basingstoke, Hants, UK). The norfloxacin disk (10 µg) was used to screen for resistance to fluoroquinolones. All isolates of *Staphylococcus aureus* and coagulase-negative *Staphylococcus*, except for *S. epidermidis*, with cefoxitin zone sizes ≤ 22 mm and all *S. epidermidis* with cefoxitin zone sizes ≤ 25 mm were found to be methicillin-resistant as confirmed by the detection of the *mecA* gene through WGS.

#### 2.4.3. Broth Microdilution Test

Minimum inhibitory concentration (MIC) determination for vancomycin was performed by broth microdilution (BrCAST/EUCAST, 2020). Solutions were prepared in Müeller–Hinton (MH) broth with adjusted concentrations of calcium (Ca^2+^) and magnesium (Mg^2+^) cations (Oxoid). Then, a final volume of 100 μL of each dilution was dispensed into 96-well polystyrene microplates. In each microdilution plate, column 11 was used as a bacterial growth control and column 12 was used as a sterility control for the medium. After the inoculum preparation, an additional dilution was performed to obtain a 10 × 10^8^ CFU/mL inoculum. A volume of 100 μL of this bacterial suspension was added to the microdilution plates already containing 100 μL of the antibiotic solution (dilution 1:2). Additionally, the test Sensititre^TM^ Gram Positive MIC Plate (Thermo Scientific^TM^, Delaware, USA) was used, which contains 16 antimicrobials, including oxacillin, levofloxacin, tigecycline, linezolid and gentamicin. A 10 μL aliquot of the 0.5 McFarland suspension was transferred to a tube containing 11 mL of MH cation broth mixed and slowly vortexed to obtain a final bacterial concentration around 2.5 × 10^8^ CFU/ ml. After homogenization, 100 μL of this bacterial suspension was inoculated into each well of the microdilution plate in broth containing the lyophilized antimicrobials, according to the manufacturer’s recommendations. The plates were incubated in an incubator at 35 °C ± 2 °C for 18–24 h; the MICs were determined as the lowest concentration of antimicrobial capable of inhibiting bacterial growth.

#### 2.4.4. Episillometric Test (E-Test)

To evaluate the MICs of rifampicin and tetracycline, the episillometric test with gradient tape (E-test^®^, BioMérieux, France) was performed, due to the lack of these salts in our laboratory during the COVID-19 pandemic period. With the aid of a swab, the sample containing 1 to 2 × 10^8^ CFU/mL was seeded on the surface of the agar plate. After an average of 15 min, the Etest^®^ strips were dispensed onto the MHagar, in 150 mm diameter plates. After the incubation period, the MIC determination was read as the intersection point between the Etest^®^ strip and the zone of inhibition of the growth of the microorganism.

### 2.5. Quantitative Assay of Biofilm Formation on Abiotic Surfaces

The assay for quantification of biofilm mass was performed as described by Stepanović et al. [21] with minor modifications. Cultures were grown for 24 h in Trypticase Soy Broth (TSB) and then bacterial suspensions were sub-cultivated in fresh TSB containing 1% glucose (TSBG) to approximately 1 × 10^8^ CFU/mL (0.5 McFarland scale) in a 96-well polystyrene plate with a flat bottom, where they were incubated for 24 h at 37 °C. The plates were washed with 0.9% saline solution three times and fixed with methanol PA, being later stained with 2% Hucker’s Crystal Violet solution. After this step, the plates were washed with running water and allowed to dry at room temperature. The absorbance reading was performed using the Synergy H1 Hybrid Multi-Mode Microplate Reader spectrophotometer (Biotek, Winooski, VT, USA). The optical density (OD) was measured at 550 nm. All isolates were tested in triplicate in three independent experiments. The results were evaluated using the scale described by Stepanovic et al. [21], by which the isolates can fit into four categories: non-biofilm producer, weak biofilm producer, intermediate biofilm producer, and strong biofilm producer. Based on the values of OD and the cut-off value (OD c), defined as three standard deviations (SD) above the mean OD of the negative control: OD c = mean OD of the negative control + (3 × SD of the negative control). The strength of biofilm production of each isolate was calculated as follows: OD ≤ OD c = non-biofilm producing, OD c < OD ≤ 2 × OD c = weak biofilm producer, 2 × OD c < OD ≤ 4 × OD c = intermediate biofilm producer and 4 × OD c < OD = strong biofilm producer.

### 2.6. Whole-Genome Sequencing and Assembly

Ten isolates of *Staphylococcus* spp. were chosen (from OIAI patients with treatment failure) for the execution of complete genome sequencing. DNA from the isolates was extracted used using the QIAamp DNA minikit (Qiagen, Hilden, Germany), according to the manufacturer’s instructions, and quantified in a Qubit Ò 3.0 fluorometer (ThermoFisher Scientific, Waltham, MA, USA). DNA libraries were prepared with the Nextera XT library prep kit (Illumina, San Diego, CA, USA) and sequenced on the Illumina HiSeq 2500 platform. The assembly and annotation of the genomes were carried out on the PATRIC (https://www.patricbrc.org/ Accessed in June 2020) platform version 3.6.12 using Unicycler v0.4.9 as a pipeline, the SPAdes v3.11.1 program optimizer and the RAST tool program [22]. The genomes were analyzed following pipelines available on the Center for Genomic Epidemiology (CGE (https://www.genomicepidemiology.org/ Acessed in June 2020)) research website, which includes a cassette of resistance (ResFinder) and virulence (VirulenceFinder) genes, allowing identification of the staphylococcal chromosome *mec* (Scc*mec*) (SccmecFinder), spa typing in *S. aureus* (SpaTyper) and Multi Locus Sequence Typing (MLST). In addition, the CARD RGI program was used for the analysis of resistance genes. Likewise, virulence genes were also searched in the Virulence Factors of Pathogenic Bacteria (VFDB) platforms. Manual curation was also carried out using SnapGene and NCBI BLAST tool to confirm the results indicated by the pipelines. Sequence Type Confirmation was done in the Public Molecular Typing Database (PubMLST), and taxonomic confirmation in the Ribosomal Multilocus Sequence Types Database (rMLST/ Institut Pauster) [23].

## 3. Results

### 3.1. Clinical Data and Bacterial Identification

#### 3.1.1. Clinical Data and MALDI-TOF MS

Of the ten patients with OIAI studied, six were male. The most frequent preoperative diagnoses were osteoarthrosis (*n* = 4) and closed fractures (*n* = 3). Prosthetic joint infection (PJI) of hip (*n* = 4) and knee (*n* = 1) were the main cause of OIAI. Demographic, clinical, and comorbidity data, preoperative diagnosis, implant type, insertion site and empirical antibiotic therapy are summarized in Table 1. Identification by MALDI-TOF MS revealed *S. aureus* (*n* = 3), *S. epidermidis* (*n*= 2), *S. haemolyticus* (*n* = 2), *S. lugdunensis* (*n* = 1), *S. sciuri* (*n* = 1), and *S. capitis* (*n* = 1). 

#### 3.1.2. Multi Locus Sequence Typing (MLST) Analysis

The GC content and genome size of the 10 Staphylococcus spp. isolates ranged from 32% to 33.7%, 2,407,407 bp to 2,894,360 bp, respectively (Table S1). The classification of S. aureus isolates according to the description of ST (sequence typing) is based on the nucleotide sequence at seven loci (arcC, aroE, GlpF, gmk, pta, pi and yqiL). The three S. aureus isolates sequenced belonged to two distinct STs, ST5 (215 and 260) and ST105 (371), all grouped into the same clonal complex (CC)-CC5, revealing a common origin. The spa type t002 was identified in two S. aureus isolates (260 and 371). The two *S. epidermidis* isolates showed two different types of STs, the 216-isolate belonging to ST2/CC2 and the 403- isolate belonging to a new ST similar to ST183. S. haemolyticus 53 and the 95 belonged to the ST9 and ST3, respectively. S. lugdunensis belonged to ST2/CC2. It was not possible to establish the STs of the isolates belonging to S. sciuri (226) and S. capitis (160), due to the unavailability of an MLST scheme in the databases used (PubMLST and CGE) (Table 2).

### 3.2. Antibiotic Susceptibility

#### 3.2.1. Phenotypic Results

Antibiotic sensitivity patterns, phenotypic and genotypic characteristics of MRSA and coagulase-negative *Staphylococci* isolates are described in Table 3 and Table 4, respectively. Briefly, the three strains of *S. aureus* were resistant to the cefoxitin disk (MRSA) and harbored *mecA* gene as confirmed by WGS. The MRSA (215) was resistant to clindamycin, levofloxacin, and gentamicin, whereas 260 expressed higher MICs for levofloxacin and gentamicin and *S. aureus* 371 for clindamycin and levofloxacin. All strains were sensitive to sulfamethoxazole + trimethoprim, linezolid and rifampicin. Vancomycin MICs (broth microdilution) of *S. aureus* ranged from 0.5 to 1.0 μg/mL. Additional MICs values for oxacillin, clindamycin, erythromycin, levofloxacin, linezolid, gentamicin, tigecycline, tetracycline and rifampicin are summarized in Table 3. 

Five of the seven isolates in the CoNS group (*S. epidermidis* (216), *S. capitis* (160) and *S. sciuri* (226), and two isolates of *S. haemolitycus* (53 and 95) showed disc resistance to cefoxitin and also harbored the *mecA* gene. Importantly, the *S. epidermidis* 216 was a multidrug-resistant strain (MDR) with higher MICs for clindamycin, levofloxacin, gentamicin, trimethoprim, rifampicin and vancomycin (MIC = 4.0 μg/mL). Conversely, the *S. epidermidis* 403 was only resistant to gentamicin. While *S. haemolitycus* 53 was resistant to tetracyclines, and gentamicin, isolate 95 was a multidrug-resistant strain (MRSH) with higher MICs also for clindamycin, levofloxacin, tetracyclines, gentamicin, and trimethoprim. *S. capitis* (160) was another MDR strain showing resistance to clindamycin, erythromycin, levofloxacin, tigecycline, and gentamicin. Resistance to clindamycin, and levofloxacin was observed in *S. sciuri* (226) and *S. lugdunensis* (167) was sensitive to all antibiotics tested. The MICs values for CoNS are summarized in Table 3.

#### 3.2.2. SCCmec Analysis and Detection of Resistance Genes by WGS

The presence of the *mecA* gene and characterization of *SCCmec* were evaluated by genome sequencing. CGE pipelines were defined for a lower limit of 90% identity and a lower bound of 60% gene sequence length. Overall, the *mecA* gene was absent only in the *S. epidermidis* (403) and *S. lugdunensis* (167) isolates. The *S. aureus* isolates 215, 260 and 371 contained the *SCCmec* type I (1B), II (2A) and V(5C2), respectively. Based on gene prediction for CoNS isolates, the *SCCmecs* detected with the highest homology were *SCCmec* type III for the *S. epidermidis* isolate (416), *SCCmec* type I and V for *S. haemolyticus* isolates (53 and 95), *SCCmec* type III for *S. sciuri* (226) and *SCCmec* type V for *S. capitis* (160) (Table 2 and Table 4).

In general, a higher frequency of antibiotic resistance genes was identified for *S. aureus* and *S. epidermidis* isolates. Regarding the genotypic profile of antibiotic resistance, all *S. aureus* isolates (215, 260 and 371) had the *mecA* and *blaZ* genes, which confer phenotypic resistance to β-lactam antibiotics. Additionally, several genes of aminoglycoside modifying enzymes (AMEs) were found, such as *aph* (3′)*-III*, *aac* (6′)*-aph* (2″), *ant* (9)*-Ia*, *ant *(4)*-Ib*, and *ant* (6)*-Ia*, and for quinolones, 260 also carried resistance genes for tetracycline, quinolones and trimethoprim (*dfrC*). The *ermA* gene that confers cross-resistance to macrolides, lincosamides and streptogramin B was also detected. Several mutations related to the quinolone resistance profile were observed in the *S. aureus* isolates (CC5). Six mutations in the *gyrA* gene, and two in the *parC* gene.

Among CoNS, *S. epidermidis* (216 and 403), *S. haemolitycus* (53 and 95), *S. lugdunensis* (167), *S. sciuri* (226) and *S. capitis* (160) were sequenced, and the blaZ gene was detected in two isolates of *S. haemolitycus* (53 and 95) and *S. capitis* (160). AME genes such as *aadD* were detected in the 216 and 53 isolates and the *aac* (6′) gene was detected in the 160 and 95 isolates. The *ermC* gene that confers cross-resistance to macrolides, lincosamides and streptogramin was detected in isolates 216 and 160 that correspond to the species *S. epidermidis* (ST2/CC2) and S. capitis, respectively (Table 4).

### 3.3. Biofilm Analysis

#### 3.3.1. Biofilm Assay

Biofilm formation in vitro in polystyrene microplates occurred for most isolates, except for *S. haemolitycus* (95) and *S. lugdunensis* (167), which did not form biofilms. *S. capitis* (160) was characterized as a weak biofilm producer, while the MRSA isolates (215, 260 and 371), *S. haemolitycus* (53), *S. epidermidis* (216 and 403), and *S. sciuri* (226) were strong biofilm formers (Table 5).

#### 3.3.2. Detection of Biofilm-Forming Genes and Adhesins (MSCRAMMs)

Table 5 describes the adhesion-related virulence genes and genes involved in toxin production found in the sequenced *Staphylococci* isolates. All isolates of *S. aureus* (215, 260, and 371) harbored genes that express surface proteins (MSCRAMMs) that facilitate adhesion, the initial step of virulence. Most genes detected were *atl* (autolysin), elastin-binding protein (*ebh* and *ebp*), *eap/map* (extracellular adhesion protein/MHC analog protein), *fnbA and fnbB* (fibronectin binding proteins), *sdrC, sdrD* and *sdrE* (fibrinogen binding proteins rich in Ser-Asp), *cflA* and *cflB* (clumping factors A and B), *ebpS* (elastin binding proteins) and *spa* (*Staphylococcal* protein A) gene, which is an important virulence factor enabling *S. aureus* to evade host immune responses.

As for the biofilm formation capacity, the complete *icaADBCR* operon (intracellular adhesins) was found in all isolates, while *IS256* was found in the 215 and 260 isolates.

Multiple toxin genes were found in *S. aureus* isolates, as summarized in Table S2. No genes encoding *pvl* and *tsst-1* were found. 

The genomic analysis of CoNS isolates (216, 403, 53, 95, 160, 226, and 167) allowed the detection of high variability in adherence genes, the most common genes being *atl*, *ebp*, and *sdr*. Except for *S. haemolitycus*, which does not harbor the *ica* locus the *S. sciuri, S. epidermidis* (216, 403) and *S. capitis* isolates carried the *icaADBCR* operon. Only the *S. epidermidis* isolates (216 and 403) contained the *sdrF*, *sdrG* and *sdrH* (Ser-Asp-rich fibrinogen binding proteins) genes. The *S. haemolitycus* isolates (53 and 95) contained only two adhesion genes, *atl* and *ebp*. In *S. capitis* (160) it was possible to identify the *atl, ebh, and ebp* genes, in addition to the *ica ADBCR* operon, the *IS*256 and, *sdrH* genes, even though this isolate was a weak biofilm former. Interestingly, in *S. sciuri* (226) was a strong biofilm former, but lack the *ica* operon and the presence of any adhesive gene. In *S. lugdunensis* (SS167) the genes *atl, ica A*, *icaB, icaC* and *icaD*, were identified.

Few virulence and enzymes producing genes were identified in CoNS isolates, the most common being *nuc* (thermonuclease), *hlb* (beta-hemolysin), *lip* (lipase) and *sspA* (serine V8 protease). The *S. epidermidis* isolates (216 and 403) contained *the sspA, sspB, geh, lip, nuc* and *hlb* genes. In *S. capitis* (160) and *S. lugdunensis* (167) only the *nuc, hlb* genes were identified. In the *S. haemolitycus* isolates (53 and 95), only the *lip, and nuc* genes were identified. In the *S. sciuri* isolate (226), only the *sspA* gene was identified. Table S3 summarizes the genes of virulence detected in the sequenced CoNS.

## 4. Discussion

In this study, we analyzed the complete genome sequencing of ten *Staphylococci* isolates from the SF of implants removed from patients with poor outcome (failure) following surgical and antibiotic treatment of OIAI (PJI and FRI). The main focus was to provide insights into the association of clinical *S. aureus* and CoNS isolates causing OIAIs with poor outcomes and specific phenotypic and genomic signatures. A growing body of evidence has pointed towards specific bacterial resistance, biofilm formation, adhesins and toxins genes of *S. aureus* and CoNS isolates [24]. 

The three MRSA isolates showed similar phenotypic and genomic characteristics concerning the pattern and presence of many antibiotic resistance (AR), biofilm formation and virulence genes, and genomic similarity (CC5) revealing a probable common origin. Interestingly, our results confirmed a previous study that identified a trend toward a higher incidence of MRSA, gentamicin resistance and hemolysis activity associated with OIAI cases [25].

The species of CoNS (*S. epidermidis, S. haemolyticus, S. lugdunensis, S. sciuri, S. capitis*) showed an expected diversity in resistance and virulence patterns, indicating the complexity of a growing number of *Staphylococci* causing OIAIs. *S. aureus* and CoNS share the skin and mucous membranes colonization niches, enabling horizontal gene transmission (HGT) of several genes along with mobile genetic elements encoding for AR, biofilm formation and virulence [26,27]. Therefore, this study adds to the importance of evaluating the phenotypic and genomic characteristics of clinical isolates of CoNS together with *S. aureus*, since there is a paucity of genomic studies on emerging species of CoNS likely to cause OIAI [28]. Indeed, despite the remaining predominance of *S. aureus* and *S. epidermidis* in the classical etiology of OIAIs, recent studies depict the increasing identification of other *Staphylococci*, including *S. haemolyticus, S. saprophyticus, S. lugdunensis, S. sciuri, S. capitis*, *and S. hominis* [27,28]. In a study by Lourtet-Hascoët et al. [29], of 215 CoNS PJI, 40% of the species were not *S. epidermidis*, with *S. capitis* and *S. lugdunensis* being the commonest.

Methicillin resistance associated with the expression of the *mecA* gene, which encodes the altered penicillin-binding protein PBP2a, may be an important factor in treatment failure in OIAI [30,31]. In the study by Post et al. [32], methicillin resistance was associated with OIAI poorer outcomes. In the current study, all *S. aureus* isolates were MRSA and belonged to CC5. The prospective cohort study published by Muñoz-Gallego et al. [33] shows a high frequency of MRSA, with 80% CC5 in PJI associated with treatment failure. In fact, MRSA isolates of hospital origin belonging to CC5 and CC8 are closed related, and spreads globally including within Latin American countries [17,34,35]. Likewise, in the CoNS group, only one strain of *S. epidermidis* (403) and *S. lugdunensis* did not harbor *mecA* gene. Despite its known pathogenicity resembling *S. aureus* life-threatening infections such as bacteremia endocarditis and PJI, carriage *mecA* gene by *S. lugdunensis* is unlikely as they remain remarkably sensitive to most antibiotics, particularly β-lactams, contrary to other members of CoNS group [36]. Although unlikely to draw any strong conclusion, our preliminary results corroborate studies that mecA gene carriage may be used as a reliable marker for Staphylococci OIAIs poor outcome [27]. 

Beyond methicillin resistance, MDR was a common feature of all of our S. aureus and S. epidermidis isolates. Except for *S. epidermidis* (403) which carried resistance genes for quinolones, rifampicin, trimethoprim, and fosfomycin, but phenotypic resistance was observed only against gentamycin. All S. aureus and S. epidermidis (216) isolates were resistant to at least three different classes of antibiotics, and resistance to gentamicin was observed in 70%, which was associated with the presence of *aac* (6′)-*Ie*-*aph* (2″)-*Ia*, *aph* (3′)-*IIIa*, *aad*D, *ant* (4′)-*Ib*, *ant* (9′)-*Ia* genes (aminoglycoside-modifying enzymes). Studies with genomic analysis of *Staphylococcus* spp. causing OIAIs have identified a strong relationship between the presence of aminoglycoside resistance genes, biofilm formation genes, and treatment failure of OIAIs [25,32]. According to Arciola et al. [11], up to 40% of *S. epidermidis* and 32% of *S. aureus* isolated from postoperative OIAIs were resistant to gentamicin. The direct relationship between aminoglycoside resistance and treatment failure in *Staphylococci* IOAI is becoming increasingly clear in recent literature, and the present study pinpoints this feature [37]. Notably, HGT occurring among staphylococcal species has been frequently evidenced, in which CoNS act as reservoirs of genes with many resistant phenotypes related to multi-resistant genes located on mobile genetic elements [27]. 

Analysis of *SCCmec* types in *S. aureus* isolates identified types I, II, and V. *SCCmec* I and II are large genetic elements capable of conferring resistance to many antibiotics and are found in hospital-sourced MRSA isolates limiting antibiotic therapy options. On the other hand, *SCCmec* V are minor genetic elements, harboring few resistant genes and are generally associated with MRSA isolates of community origin [35,38]. Interestingly, the three MRSA isolates were recovered from sonication fluid of osteosynthesis from patients with treatment failure associated with FRI (260 and 371) and spinal infection (215) in which ciprofloxacin had been employed empirically, while cefazolin was used to patients with S. epidermidis (216) and *S. lugdunensis* infection, and vancomycin to the others. We argue that the antibiotic selective pressure may have influenced the antibiotic resistant patterns and also in the patient’s poor outcomes.

The isolate 260 with *SCCmec* V was sensitive to clindamycin and quinolone, unlike the isolates with *SCCmec* I and II (215 and 371). The reported cases of community-acquired MRSA (CA-MRSA) bone infections are infrequent and predominantly described in hematogenous osteomyelitis of long bones in children younger than two years of age [39].

In the sequencing data of our *S. aureus*, *S. epidermidis, S. lugdunensis* and *S. capitis* isolates, the *icaADBC* operon was found. Besides, *IS256* gene was also identified in the *S. aureus* (215, 260), *S. epidermidis* (216), *S. haemolyticus* (95) and *S. capitis*. ST2 clonal type always bears the insertion sequence *IS256* and *ica* genes, which are implicated in biofilm production [24]. We hypothesize that the co-presence of both virulence factors in addition to *mecA* gene may be labeled as possible genomic makers also for non-epidermidis CoNS causing OIAI with poor outcome. *S. epidermidis* and *S. lugdunensis* harboring, respectively, *ica* and IS256 genes, have been identified in pathogenic strains associated with severe infections such as endocarditis and OIAIs [36]. Moreover, the presence of these three markers (*icaA*, *mecA* and *IS256*) has been significantly associated with CoNS invasive nosocomial strains [40].

In our study, the *S. epidermidis* (216) isolate belonging to the ST2 (CC2), showed a strong biofilm production, harbored multiple genes associated with adherence, to host matrix binding proteins (*atl, ebh, clfA*), to biofilm formation (*icaADBC* locus and *IS256* gene), resistance to rifampicin, and had a MIC of 4 μg/mL for vancomycin. Interestingly, it was isolated from a patient with a history of diabetes and a bone tumor, with a closed fracture of the femur that progressed to FRI who had previously received vancomycin and rifampicin as chronic suppression therapy for 12 weeks. Epidemic isolates of CoNS ST2 and ST5 have been isolated worldwide, including in Brazil, and are likely related to the presence of the *cfr* gene that confers resistance to linezolid, are associated with musculoskeletal infections (PJI, FRI) [41,42]. Sanchéz et al. [1] reported that pathogenic isolates of *S. epidermidis* causing PJI often belong to the ST2 clone and carry genes such as the *ica* operon, *IS256, sdrF, bhp* and *mecA*. Indeed, the presence of the *ica* operon in *S. epidermidis* isolates has long been associated with biofilm production [42]. 

Furthermore, in the genomic analysis of the non-epidermidis CoNS isolates (*S. lugdunensis, S. capitis, S. lugdunensis, and S. sciuri*) we aimed at searching for possible virulence markers in the OIAI setting due to the scarcity of literature. Despite the great variability in the presence of genes that express adherence proteins (MSCRAMMs) and other virulence traits, the most frequently identified genes were *atl*, *ebp*, *hlb* and *nuc*. However, in the *S. sciuri* isolate we were unable to find any genomic marker towards adhesion or biofilm-forming gene, despite harboring *mecA* gene and having shown a strong capacity for biofilm formation. Interestingly, the animal origin *S. sciuri* species group have been proposed as the origin and/or reservoir of the *S. aureus mecA* gene, which has been increasingly identified as a OIAI etiology [3,43]. This isolate was identified in a patient with bone tumor who underwent endoprosthetic reconstructions for lower limb savage and evolved to infected endoprostheses. Treatment consisted of multiple surgeries of debridement, antibiotics as suppressive therapy and implant retention. Future genomic studies are needed to better understand this pathogen in OIAIs.

*S. haemolyticus* isolates carried few adherence genes (*atl* and *ebp*). They have been frequently associated with MDR strain outbreaks in the hospital setting including neonatal units, but currently are the second most frequent CoNS in implant-associated infections. They are reported to have the highest level of antibiotic resistance, which seems to be the main genomic marker for this CoNS [27,43]. In fact, our *S. haemolyticus* strains carried multiple AR traits, including resistant genes for β-lactam, aminoglycosides, tetracyclines, quinolones, and clindamycin. Nevertheless, few publications have completely assessed the virulence traits in this species, warranting future studies [24].

Conversely, our *S. capitis* is an MDR isolate (resistance to β-lactam, AME, MLS_b_ and quinolones) and had several biofilm-forming (complete *ica* operon and *IS256*), adherence (*atl, ebh, ebp, sdrE*) and other virulence (*hlb* and *nuc*) genes. The genomic analysis of this isolate adds importance due to the scarcity of clinical reports on OIAIs caused by *S. capitis*. Recently, genomic sequencing analysis of a large amount of *S. capitis* collection including OIAI strains was carried out by Swedish researchers. They identified a robust biofilm-forming ability and MDR traits and made speculations regarding the in-hospital dissemination of this pathogen that has been classically associated with neonatal intensive care unit sepsis [44]. MDR is likely a crucial issue in *S. capitis* infection, in which our isolate expressed an MDR phenotype and a vancomycin MIC = 2.0 µg/mL. 

Despite harboring no resistance genes, our *S. lugdunensis* isolate (ST2/CC2) had *ica*ABC operon and *atl, sdrC, hld* and *nuc* genes. *S. lugdunensis* produces a fibrinogen-binding protein linked to the bacterial cell wall that has been involved with endocarditis and persistent bacteremia in vitro studies [36]. The presence of *ica* operon and *atl* gene (autolysin) in biofilm formation may play a role in the OIAI poor outcomes in patients.

We acknowledge that the analysis of very small number of isolates is a strong limitation, and few conclusions can be drawn. The importance of this type of research would be at assessing specific traits including bacterial resistance, biofilm formation, adhesins and toxins genes of *S. aureus* and CoNS isolates that would trigger preventive measures to be taken at the bed site before carrying out elective surgeries. Moreover, the molecular epidemiology of non-epidermidis CoNS implant-associate infection isolates need more attention. The literature regarding this type of research has been focusing on *S. aureus* and *S. epidermidis*, but scarce among other CoNS. Besides, back no more than a decade ago, new microbiological technologies including the use of MALD-TOF MS was a tool to be applied only to few universities’ microbiological laboratories. However, it is currently widespread available, including in developing countries’ public hospitals. We also envision that metagenomics and WGS technology may assume the same importance in the near future, which may change the way we apply microbiology at the bed site to prevent, diagnosis and treat bacterial infections. The ordinary medical strategies that have been carried out today in the daily basis may have little impact to the genomic analysis of pathogens storage at the microbiological laboratory. However, in many implant-associated and orthopaedic infections, the phenotypic bacterial resistance identification does not help either in the management of this infection. An important number of these infections is due to the presence of pathogens growing into biofilms, in which the traditional approach of prescribing antibiotics based upon phenotypical susceptibility testing to determine the adequate therapeutic approach is unhelpful. Understanding the bacterial behavior of emerging pathogens in this medical situation seems to be crucial. Moreover, the retrospective analysis of patients’ records accounts for the unknown exact number of tissue samples collected per patients. However, the isolates came from sonication fluid from implants taken from patients with PJI and FRI, conferring a broad spectrum of IOAIs. Finally, we were unable to carry out rifampicin and tetracycline sensitivity using the gold standard technique of broth microdilution due to the lack of these salts in our laboratory during the COVID-19 pandemic period. To overcome this situation, the episillometric test with gradient tape (E-test®) was carried out. Genomic evaluation of *S. aureus* isolates together with CoNS may seem confusing, but these comparisons with the clinical outcomes can draw attention to common bacterial sources and the transfer of antibiotic resistance genes between these species.

## 5. Conclusions

In conclusion, the genomic analysis of *Staphylococci* allowed elucidation of MRSA and CoNS features that are associated with treatment failure in OIAIs. Internationally spreading isolates of MRSA and *S. epidermidis* are associated with OIAI in Brazil. The MRSA isolates showed genomic similarity, revealing a probable common origin, and harboring multiple resistance, biofilm formation, and virulence genes. Our findings corroborate a probable association between isolates harboring resistance genes to β-lactam and aminoglycosides with treatment failure in OIAIs. In addition to *S. epidermidis*, there is variability in CoNS isolates with distinct genomic features that require further attention.

## Figures and Tables

**Table 1:** Clinical data of patients with infections associated with orthopedic implants.

Patient	Age	Gender	Comorbidities	Preoperative Diagnosis	Source	Implant	Bacterial Identification (MALDI-TOF MS)	Empirical Antibiotic Therapy
215	38	M	DM	Chronic spinal disease	Spine	Plate/screw	*S. aureus*	Ciprofloxacin
260	89	M	DM and Tumor	Closed fracture	tibia/fibula	Plate/screw	*S. aureus*	Ciprofloxacin
371	43	M		Closed fracture	ankle	Plate/screw	*S. aureus*	Ciprofloxacin
216	47	M	DM and Tumor	Closed fracture	hip	Plate/screw	*S. epidermidis*	Cefazolin
403	67	M	_	Osteoarthrosis	hip	Arthroplasty	*S. epidermidis*	Vancomycin
53	66	M	_	Osteoarthrosis	hip	Arthroplasty	*S. haemolyticus*	Vancomycin
95	67	F	DM and Coronariopathy	Osteoarthrosis	knee	Arthroplasty	*S. haemolyticus*	Vancomycin
160	53	F	RA	Osteoarthrosis	hip	Arthroplasty	*S. capitis*	Vancomycin
226	44	F	_	Tumor lesion	hip	Arthroplasty	*S. sciuri*	Vancomycin
167	79	F	Tumor	Open fracture	ankle	Fixing pin	*S. lugdunensis*	Cefazolin + Gentamicin

F: female gender; M: male gender; DM: diabetes melitus; RA: rheumatoid arthritis; MALDI-TOF MS: Matrix-assisted laser desorption/ionization time-of-flight mass spectroscopy.

**Table 2:** Molecular epidemiology of *Staphylococcus* spp. clinical isolates included in the study.

Isolate ID	Species	Molecular Characterization			
		**SCC*mec*** **Type**	**ST**	**Clonal Complex**	**GeneBank Number**
215	*S. aureus*	I (1B)	5	CC5	JAHMMM000000000
260	*S. aureus*	V (5C2)	5	CC5	JAHMMN000000000
371	*S. aureus*	(2A)	105	CC5	JAHMMO000000000
216	*S. epidermidis*	III (3A)	2	CC2	JAHMMP000000000
403	*S. epidermidis*	_	183	_	JAHMMQ000000000
53	*S. haemolyticus*	_	9	_	JAHMMR000000000
95	*S. haemolyticus*	_	3	_	JAHMMS000000000
160	*S. capitis*	_	_	_	JAHMMT000000000
226	*S. sciuri*	III (3A)	_	_	JAHMMU000000000
167	*S. lugdunensis*	_	2	CC2	JAHMMV000000000

CC Clonal Complex; SCC*mec* Staphylococcal Cassette Chromosome *mec*; ST Sequence Type.

**Table 3:** Phenotypic resistance profile using disk diffusion test, and broth microdilution and E-test for minimal inhibitory concentration (MIC) characterization of *Staphylococcus* spp. isolates included in the study.

ID	Species	MIC									
		Broth Microdilution								E-Test	
		(µg/mL)									
		VAN	OXA	CLI	ERY	LEV	LNZ	GEN	TGC	TET	RIF
215	*S. aureus*	1.0	>2.0	2.0	<0.5	1.0	2.0	2.0	0.25	0.75	<0.016
260	*S. aureus*	0.5	>2.0	0.25	<0.5	1.0	2.0	2.0	0.25	0.19	<0.016
371	*S. aureus*	0.5	>2.0	1.0	<0.5	1.0	2.0	<1.0	0.25	0.5	<0.016
216	*S. epidermidis*	4.0	>2.0	1	<0.5	>4.0	2.0	2.0	0.25	0.094	>256
403	*S. epidermidis*	1.0	<0.25	<0.25	<0.5	<0.5	2.0	>8.0	0.25	0.125	0. 016
53	*S. haemolyticus*	0.5	>2.0	<0.25	<0.5	<0.5	2.0	2.0	2.0	24	<0.016
95	*S. haemolyticus*	1.0	>2.0	>2.0	<0.5	>4.0	1.0	>8.0	1.0	32	<0.016
160	*S. capitis*	2.0	>2.0	>2.0	>4.0	>4.0	1.0	>8.0	1.0	0.75	<0.016
226	*S. Sciuri*	1.0	>2.0	2.0	<0.5	1.0	2.0	<1.0	0.25	0.5	<0.016
167	*S. lugdunensis*	1.0	0.5	<0.25	<0.5	<0.5	0.5	<1.0	0.06	0.125	<0.016

SA *Sthapylococus aureus*; SE *Staphylococcus epidermidis*; SH *Staphylococcus haemolyticus*; SC *Staphylococcus capitis*; SSc Staphylococcus sciuri; SL Staphylococus lugdunensis; FOX Cefoxitin; OXA Oxacillin; CLI Clindamycin; ERY Erithromycin; NOR Norfloxacin; GEN Gentamicin; TET Tetracycline; RIF Rifampicin; SXT trimethoprim sulfamethoxazole; MIC: minimal inhibitory concentration.

**Table 4:** Genotypic resistance profile with respective genes of *Staphylococcus* spp. isolates included in the study.

Antibiotics	Resistance Genes	*S. aureus*			*S. epidermidis*		*S. haemolyticus*		*S. capitis*	*S. sciuri*	*S. lugdunensis*
		215	260	371	216	403	53	95	160	226	167
β-lactam	*blaZ*	✓	✓	✓			✓	✓	✓		
	*mecA*	✓	✓	✓	✓		✓	✓	✓	✓	
Aminoglycosides	*aph (3′)-III*	✓		✓							
	*aadD*			✓	✓		✓				
	*ant (4′)-Ib*	✓		✓				✓	✓		
	*aac (6′)-aph (2″)*	✓						✓	✓		
	*ant (9)-Ia*	✓		✓							
	*ant (6)-Ia*	✓									
MLSb	*erm (A)*	✓		✓							
	*erm (B)*				✓						
	*erm(C)*				✓				✓		
Tetraciclyn	*tet (38)*		✓	✓							
	*tet (K)*						✓	✓			
Quinolones	*gyrA (p.G208L)*	✓			✓						
	*gyrA (p.S84T)*	✓			✓						
	*gyrA (p.S84L)*	✓		✓							
	*gyrA (p.S80L)*	✓									
	*gyrA (p.T457A)*		✓								
	*gyrA (Xaa172Ala)*		✓			✓					
	*parC (S80Y)*			✓	✓	✓					
	*parC (E84G)*			✓	✓	✓					
Others	*sdrM*			✓	✓	✓					
	*fosB*			✓	✓	✓					
	*fusB*							✓			
	*dfrC*		✓			✓			✓		
	*rpoB (I527M)*					✓					
	*rpoB (D471E)*				✓						

MLS_b_ resistance to macrolides, lincosamides and group B streptogramins. SA *Sthapylococus aureus*; SE *Staphylococcus epidermidis*; SH *Staphylococcus haemolyticus*; SC *Staphylococcus capitis*; SSc *Staphylococcus sciuri*; SL *Staphylococus lugdunensis*.

**Table 5:** Evaluation of phenotypic biofilm formation and detection of biofilm forming genes and adhesins (MSCRAMMs) of clinical isolates.

Species ID	Biofilm Formation	PIA	Autolysin	FBP	EBP	FP	AF	ECAP/MHCAP	Sdr-FP
*S. aureus*	Strong	*icaA, icaB, icaC*, *icaD, icaR, IS256*	*atl, atlA*	*ebh*	*ebp, ebpS*	*fnbA*	*cflA, cflB*	*eap/map*	*sdrC, sdrD, sdrE*
215									
*S. aureus*	Strong	*icaA, icaB, icaC, icaD, icaR, IS256*	*atl, atlA*	*ebh*	*ebp, ebpS*	*fnbA, fnbB*	*cflA, cflB*	*eap/map*	*sdrC, sdrD, sdrE*
260									
*S.aureus*	Strong	*icaA, icaB, icaC, icaD, icaR, IS256*	*atl, atlA*	*ebh*	*ebp, ebpS*	*fnbA, fnbB*	*cflA, cflB*	*eap/map*	*sdrC, sdrD, sdrE*
371									
*S. epidermidis*	Strong	*icaA, icaB, icaC, icaD, icaR, IS256*	*atl, atlE*	*ebh*	*ebp*		*cflA*		*sdrF, sdrG/fbe, sdrH*
216									
*S. epidermidis*	Strong	*icaA, icaB, icaC, icaD, icaR*,	*atl, atlE*	*ebh*	*ebp*				*sdrF, sdrG/fbe, sdrH*
403									
*S. haemolyticus*	Strong		*atl*		*ebp*				
53									
*S. haemolyticus*	non-	*IS256*	*atl*		*ebp*				
95	adherent								
*S. capitis*	weak	*icaA, icaB, icaC, icaD, icaR, IS256*	*atl*	*ebh*	*ebp*				
160									
*S. sciuri*	Strong								
226									
*S. lugdunensis*	non-	*icaA, icaB, icaC, icaD, icaR*,	*atl*						
167	adherent								

PIA: Polyssacharide intercellular adhesin; FBP: fibronectin binding proteins; EBP: elastin binding proteins; FB: fibrinectin protein; AF: Agglutination factor; Sdr-FP: Sdr family proteins; ECA/MHC AP: Extracellular adhesion protein/MHC analog protein; MSCRAMMs Microbial Surface Components Recognizing Adhesive Matrix Molecules.

## Data Availability

The datasets presented in this study can be found in online repositories. The names of the repository/repositories and accession number(s) can be found below: https://www.ncbi.nlm.nih.gov/ Accessed on 15 October 2021, BioProject number PRJNA736948.

## Supplementary Materials

The following supporting information can be downloaded at: https://www.mdpi.com/article/10.3390/microorganisms10061149/s1: title; Table S1: Genome assembly metrics of 10 *Staphylococcus* genomes; Table S2: Virulence genes of *S. aureus* isolates sequenced by WGS; Table S3: Virulence genes of CoNS isolates sequenced by WGS.

## References

[1] Sánchez A., Benito N., Rivera A., García L., Miró E., Mur I., González Y., Gutiérrez C., Horcajada J.P., Espinal P. (2020). Pathogenesis of *Staphylococcus epidermidis* in prosthetic joint infections: Can identification of virulence genes differentiate between infecting and commensal strains?. J. Hosp. Infect..

[2] Triffault-Fillit C., Ferry T., Laurent F., Pradat P., Dupieux C., Conrad A., Becker A., Lustig S., Fessy M.H., Chidiac C. (2019). Microbiologic epidemiology depending on time to occurrence of prosthetic joint infection: A prospective cohort study. Clin. Microbiol. Infect..

[3] Becker K., Heilmann C., Peters G. (2014). Coagulase-negative staphylococci. Clin. Microbiol. Rev..

[4] Alder K., Lee I., Munger A.M., Kwon H.-K., Morris M.T., Cahill S.V., Back J., Yu K.E., Lee F.Y. (2020). Intracellular *Staphylococcus aureus* in bone and joint infections: A mechanism of disease recurrence, inflammation, and bone and cartilage destruction. Bone.

[5] Gristina A.G., Naylor P., Myrvik Q. (1988). Infections from biomaterials and implants: A race for the surface. Med. Prog. Technol..

[6] Subbiahdoss G., Kuijer R., Grijpma D.W., van der Mei H.C., Busscher H.J. (2009). Microbial biofilm growth vs. tissue integration: “the race for the surface” experimentally studied. Acta Biomater..

[7] Montanaro L., Speziale P., Campoccia D., Ravaioli S., Cangini I., Pietrocola G., Giannini S., Arciola C.R. (2021). Scenery of *Staphylococcus* implant infections in orthopedics. Future Microbiol..

[8] Chu L., Yang Y., Yang S., Fan Q., Yu Z., Hu X.-L., James T.D., He X.-P., Tang T. (2018). Preferential colonization of osteoblasts over co-cultured bacteria on a bifunctional biomaterial surface. Front. Microbiol..

[9] Wildeman P., Tevell S., Eriksson C., Lagos A.C., Söderquist B., Stenmark B. (2020). Genomic characterization and outcome of prosthetic joint infections caused by *Staphylococcus aureus*. Sci. Rep..

[10] Barbieri R., Pesce M., Franchelli S., Baldelli I., de Maria A., Marchese A. (2015). Phenotypic and genotypic characterization of Staphylococci causing breast peri-implant infections in oncologic patients. BMC Microbiol..

[11] Arciola C.R., Campoccia D., Montanaro L. (2018). Implant infections: Adhesion, biofilm formation and immune evasion. Nat. Rev. Microbiol..

[12] Jabbouri S., Sadovskaya I. (2010). Characteristics of the biofilm matrix and its role as a possible target for the detection and eradication of *Staphylococcus epidermidis* associated with medical implant infections. FEMS Immunol. Med. Microbiol..

[13] Götz F. (2002). Staphylococcus and biofilms. Mol. Microbiol..

[14] Otto M. (2012). Molecular basis of *Staphylococcus epidermidis* infections. Semin. Immunol..

[15] Trampuz A., Zimmerli W. (2006). Diagnosis and treatment of infections associated with fracture-fixation devices. Injury.

[16] Tande A.J., Patel R. (2014). Prosthetic joint infection. Clin. Microbiol. Rev..

[17] Yano M.H., Klautau G.B., da Silva C.B., Nigro S., Avanzi O., Mercadante M.T., Salles M.J. (2014). Improved diagnosis of infection associated with osteosynthesis by use of sonication of fracture fixation implants. J. Clin. Microbiol..

[18] Metsemakers W., Morgenstern M., McNally M., Moriarty F., McFadyen I., Scarborough M., Athanasou N., Ochsner P., Kuehl R., Raschke M. (2018). Fracture-related infection: A consensus on definition from an international expert group. Injury.

[19] Parvizi J., Tan T.L., Goswami K., Higuera C., Della Valle C., Chen A.F., Shohat N. (2018). The 2018 Definition of Periprosthetic Hip and Knee Infection: An Evidence-Based and Validated Criteria. J. Arthroplast..

[20] Trampuz A., Piper K.E., Jacobson M.J., Hanssen A.D., Unni K.K., Osmon D.R., Patel R. (2007). Sonication of removed hip and knee prostheses for diagnosis of infection. N. Engl. J. Med..

[21] Stepanović S., Vuković D., Dakić I., Savić B., Švabić-Vlahović M. (2000). A modified microtiter-plate test for quantification of staphylococcal biofilm formation. J. Microbiol. Methods.

[22] Brettin T., Davis J.J., Disz T., Edwards R.A., Gerdes S., Olsen G.J., Olson R., Overbeek R., Parrello B., Pusch G.D. (2015). RASTtk: A modular and extensible implementation of the RAST algorithm for building custom annotation pipelines and annotating batches of genomes. Sci. Rep..

[23] Thomsen M.C.F., Ahrenfeldt J., Cisneros J.L.B., Jurtz V.I., Larsen M.V., Hasman H., Aarestrup F., Lund O. (2016). A bacterial analysis platform: An integrated system for analysing bacterial whole genome sequencing data for clinical diagnostics and surveillance. PLoS ONE.

[24] Argemi X., Hansmann Y., Prola K., Prévost G. (2019). Coagulase-Negative Staphylococci Pathogenomics. Int. J. Mol. Sci..

[25] Post V., Wahl P., Uçkay I., Ochsner P., Zimmerli W., Corvec S., Loiez C., Richards R.G., Moriarty T.F. (2014). Phenotypic and genotypic characterisation of *Staphylococcus aureus* causing musculoskeletal infections. Int. J. Med. Microbiol..

[26] Chon J.W., Lee U.J., Bensen R., West S., Paredes A., Lim J., Khan S., Hart M.E., Phillips K.S., Sung K. (2020). Virulence characteristics of meca-positive multidrug-resistant clinical coagulase-negative staphylococci. Microorganisms.

[27] França A., Gaio V., Lopes N., Melo L. (2021). Virulence Factors in Coagulase-Negative *Staphylococci*. J. Pathog..

[28] Michels R., Last K., Becker S.L., Papan C. (2021). Update on coagulase-negative staphylococci—What the clinician should know. Microorganisms.

[29] Lourtet-Hascoët J., Félicé M.P., Bicart-See A., Bouige A., Giordano G., Bonnet E. (2018). Species and antibiotic susceptibility testing of coagulase-negative staphylococci in periprosthetic joint infections. Epidemiol. Infect..

[30] Salgado C.D., Dash S., Cantey J.R., Marculescu C.E. (2007). Higher risk of failure of methicillin-resistant *Staphylococcus aureus* prosthetic joint infections. Clin. Orthop. Relat. Res..

[31] Lora-Tamayo J., Murillo O., Iribarren J.A., Soriano A., Sánchez-Somolinos M., Baraia-Etxaburu J.M., Rico A., Palomino J., Rodríguez-Pardo D., Horcajada J.P. (2013). A large multicenter study of methicillin-susceptible and methicillin-resistant *Staphylococcus aureus* prosthetic joint infections managed with implant retention. Clin. Infect. Dis..

[32] Post V., Harris L.G., Morgenstern M., Mageiros L., Hitchings M.D., Méric G., Pascoe B., Sheppard S.K., Richards R.G., Moriarty T.F. (2017). Comparative genomics study of *Staphylococcus epidermidis* isolates from orthopedic-device-related infections correlated with patient outcome. J. Clin. Microbiol..

[33] Muñoz-Gallego I., Viedma E., Esteban J., Mancheño-Losa M., García-Cañete J., Blanco-García A., Rico A., García-Perea A., Garbajosa P.R., Escudero-Sánchez R. (2020). Genotypic and Phenotypic Characteristics of *Staphylococcus aureus* Prosthetic Joint Infections: Insight on the Pathogenesis and Prognosis of a Multicenter Prospective Cohort. Open Forum Infect. Dis..

[34] Jian Y., Li T., Zhao L., Zhao N., Liu Y., Lv H., Wang Y.N., Liu Q., Li M. (2021). Regulation of bla system in ST59-related oxacillin-susceptible mecA-positive *Staphylococcus aureus*. J. Antimicrob. Chemother..

[35] Leme R.C.P., Bispo P.J.M., Salles M.J. (2021). Community-genotype methicillin-resistant *Staphylococcus aureus* skin and soft tissue infections in Latin America: A systematic review. Braz. J. Infect Dis..

[36] Argemi X., Hansmann Y., Riegel P., Prévost G. (2017). Is *Staphylococcus lugdunensis* significant in clinical samples?. J. Clin. Microbiol..

[37] Månsson E., Johannesen T.B., Nilsdotter-Augustinsson Å., Söderquist B., Stegger M. (2021). Comparative genomics of *Staphylococcus epidermidis* from prosthetic-joint infections and nares highlights genetic traits associated with antibiotic resistance, not virulence. Microb. Genom..

[38] Hellmark B., Söderquist B., Unemo M., Nilsdotter-Augustinsson Å. (2013). Comparison of *Staphylococcus epidermidis* isolated from prosthetic joint infections and commensal isolates in regard to antibiotic susceptibility, agr type, biofilm production, and epidemiology. Int. J. Med. Microbiol..

[39] Vardakas K.Z., Kontopidis I., Gkegkes I.D., Rafailidis P.I., Falagas M.E. (2013). Incidence, characteristics, and outcomes of patients with bone and joint infections due to community-associated methicillin-resistant *Staphylococcus aureus*: A systematic review. Eur. J. Clin. Microbiol. Infect. Dis. Off. Publ. Eur. Soc. Clin. Microbiol..

[40] Conlan S., Mijares L.A., Becker J., Blakesley R.W., Bouffard G.G., Brooks S., Coleman H., Gupta J., Gurson N., Park M. (2012). *Staphylococcus epidermidis* pan-genome sequence analysis reveals diversity of skin commensal and hospital infection-associated isolates. Genome Biol..

[41] Martínez-Meléndez A., Morfín-Otero R., Villarreal-Treviño L., Camacho-Ortíz A., González-González G., Llaca-Díaz J., Rodríguez-Noriega E., Garza-González E. (2016). Molecular epidemiology of coagulase-negative bloodstream isolates: Detection of *Staphylococcus epidermidis* ST2, ST7 and linezolid-resistant ST23. Braz. J. Infect Dis..

[42] Lee A.S., de Lencastre H., Garau J., Kluytmans J., Malhotra-Kumar S., Peschel A., Malhotra-Kumar S. (2018). Methicillin-resistant *Staphylococcus aureus*. Nat. Rev. Dis. Primers.

[43] Heilmann C., Ziebuhr W., Becker K. (2019). Are coagulase-negative *staphylococci* virulent?. Clin. Microbiol. Infect..

[44] Tevell S., Baig S., Hellmark B., Simoes P.M., Wirth T., Butin M., Nilsdotter-Augustinsson Å., Söderquist B., Stegger M. (2020). Presence of the neonatal *Staphylococcus capitis* outbreak clone (NRCS-A) in prosthetic joint infections. Sci. Rep..

