# Supplementary material for: Comparative Phenotypic and Genomic Features of Staphylococci from Sonication Fluid of Orthopedic Implant-Associated Infections with Poor Outcome
Source: Microorganisms. 2022 Jun 2;10(6):1149. doi: 10.3390/microorganisms10061149 (PMC9230661; doi:10.3390/microorganisms10061149)
Supplement: Supplementary file 1 [file microorganisms-10-01149-s001.zip › microorganisms-1734856-supplementary.pdf]

Supplementary Material

Table S1: Genome assembly metrics of 10 Staphylococcus genomes.

Isolate ID	Species	Total length (bp)	GC content (%)	No. of contigs	N50 (bp)	L50 (contigs)	GeneBank Acession No.
**SS 215**	*S. aureus*	2,864,743	32.75	68	87,378	10	JAHMMM000000000
**SS 260**	*S. aureus*	2,843,999	32.73	27	347,171	3	JAHMMN000000000
**SS 371**	*S. aureus*	2,894,360	32.78	60	223,541	4	JAHMMO000000000
**SS 216**	*S. epidermidis*	2,484,686	32.15	41	160,954	5	JAHMMP000000000
**SS 403**	*S. epidermidis*	2,490,824	32.00	40	176,132	5	JAHMMQ000000000
**SS 53**	*S. haemolyticus*	2,407,407	32.73	71	78,711	9	JAHMMR000000000
**SS 95**	*S. haemolyticus*	2,663,104	32.55	97	69,426	12	JAHMMS000000000
**SS 160**	*S. capitis*	2,508,421	32.80	22	545,816	2	JAHMMT000000000
**SS 226**	*S. sciuri*	2,883,412	32.45	21	420,094	2	JAHMMU000000000
**SS 167**	*S. lugdunensis*	2,548,764	33.69	24	176,495	5	JAHMMV000000000

Table S2: Virulence genes of *S. aureus* isolates sequenced by WGS.

			**Isolate (ID)**			**Reference strain**
**Class**	**Virulence Factor**	**genes**	**215**	**2260**	**371**	***S.aureus* RF122**
**Adhesion**						chromosome (NC_007622)
	Autolysine	***atl***	+	+	+	SAB0919c
	Cell wall-associated fibronectin binding protein	***ebh***	+	+	+	SAB1289c*
	Agglutination factor A	***clfA***	+	+	+	SAB0744*
	Agglutination factor B	***clfB***	+	+	+	SAB2505c
	Collagen adhesion	***cna***	-	-	-	-
	Elastin binding protein	***ebp***	+	+	+	SAB1343c
	Extracellular adhesion protein / MHC analog protein	***eap/map***	+	+	+	SAB1873c
	Fibrinogen binding protein	***efb***	-	+	+	SAB1021
	Fibronectin binding proteins	***fnbA***	+	+	+	SAB2375c
		fnbB	-	+	+	-
	Intercellular adhesin	***icaA***	+	+	+	SAB2541
		icaB	+	+	+	SAB2543
		icaC	+	+	+	SAB2544
		icaD	+	+	+	SAB2542
		icaR	+	+	+	SAB2540c
	Ser-Asp-rich fibrinogen binding proteins	***sdrC***	+	+	+	SAB0512*
		sdrD	+	+	+	-
		sdrE	+	+	+	SAB0513
		sdrF	-	-	-	-
		sdrG	-	-	-	-

		sdrH	-	-	-	-
	Staphylococcal protein A	***spa***	+	+	+	SAB0050c*
Toxin	Alpha hemolysin	***hly/hla***	+	+	+	*SAB1027c*
	Beta hemolysin	***hlb***	+	+	+	*SAB1874*
	Delta hemolysin	***hld***	+	+	+	-
	EnterotoxinaA	***sea***	-	-	-	-
	Enterotoxin B	***seb***	-	-	-	-
	Enterotoxin C	***sec***	-	-	-	*SAB0363*
	Enterotoxin aG	***seg***	+	+	+	*SAB1696c*
	Type A exfoliative toxin	***eta***	+	+	+	*SAB1037c*
	Exotoxin	***set/11***	+	+	+	-
		set/13	+	+	+	-
		set/15	+	+	+	-
		set/37	+	+	+	-
		set/39	+	+	+	-
		set/06	+	+	+	-
		set/07	+	+	+	-
		set/08	+	+	+	-
		set/09	+	+	+	-
	Gamma hemolysin	***hlgA***	+	+	+	*SAB2300*
		hlgB	+	+	+	*SAB2302*
		hlgC	+	+	+	*SAB2301*
	Leukocidin M	***lukF-like***	-	-	-	*SAB0783*
		lukM	-	-	-	*SAB0782*
	Leukocidin D	***lukD***	+	+	+	*SAB1686c*
	Leukocidin E	***lukE***	-	-	-	*SAB1687c*
	Leucocidina Panton-Valentine<br>Panton-Valentine Leukocidin	***lukF-PV***	-	-	-	-
		lukS-PV	-	-	-	-
	Toxic shock syndrome toxin	***tsst***	-	-	-	*SAB0360c*

Table S3: Virulence genes of CoNS isolates sequenced by WGS

			Isolate (ID)						
**Classe**	**Fator de virulência**	**genes**	**216**	**403**	**53**	**95**	**160**	**226**	**167**
**Adhesion**	Autolysin	***atl***	+	+	+	+	+	-	+
	Cell wall associated fibronectin binding protein	***ebh***	+	+	-	-	+	-	-
	Clumping factor A	***clfA***	+	-	-	-	-	-	-
	Clumping factor B	***clfB***	-	-	-	-	-	-	-
	Collagen adhesion	***cna***	-	-	-	-	-	-	-
	Elastin binding protein	***ebp***	+	+	+	+	+	-	-
	Extracellular adherence protein/MHC analogous protein	***eap/map***	-	-	-	-	-	-	-
	Fibrinogen binding protein	***efb***	-	-	-	-	-	-	-
	Fibronectin binding proteins	***fnbA***	-	-	-	-	-	-	-
		fnbB	-	-	-	-	-	-	-
	Intercellular adhesin	***icaA***	+	+	-	-	+	-	+
		icaB	+	+	-	-	+	-	+
		icaC	+	+	-	-	+	-	+
		icaD	+	+	-	-	-	-	+
		icaR	+	+	-	-	+	-	+
	Ser-Asp rich fibrinogen-binding proteins	***sdrC***	-	-	-	-	-	-	-
		sdrD	-	-	-	-	-	´-	-
		sdrE	-	-	-	-	-	-	-
		sdrF	+	+	-	-	-	-	-
		sdrG	+	+	-	-	-	-	-
		sdrH	+	+	-	-	-	-	-

	Staphylococcal protein A	***spa***	-	-	-	-	-	-	-
Enzyme	Serine V8 protease	**sspA**	+	+	-	-	-	+	-
	Cysteine protease	***sspB***	+	+	-	-	-	-	-
		sspC	-	-	-	-	-	-	-
	Hyaluronate lyase	***hysA***	-	-	-	-	-	-	-
	Lipase	***geh***	+	+	-	-	-	-	-
		lip	+	+	+	+	-	-	-
	Serine V8 protease	***sspA***	+	+	-	-	-	-	-
	Staphylocoagulase	***coa***	-	-	-	-	-	-	-
	Thermonuclease	***nuc***	+	+	+	+	+	-	+
Toxin	Alpha hemolysin	***hly/hla***	-	-	-	-	-	-	-
	Beta hemolysin	***hlb***	+	+	-	-	+	-	+
	Enterotoxin A	***sea***	-	-	-	-	-	-	-
	Enterotoxin B	***seb***	-	-	-	-	-	-	-
	Enterotoxin C	***sec***	-	-	-	-	-	-	-
	Enterotoxin D	***sed***	-	-	-	-	-	-	-
	Exfoliative toxin type A	***eta***	-	-	-	-	-	-	-
	Exfoliative toxin type B	***etb***	-	-	-	-	-	-	-
	Exfoliative toxin type C	***etc***	-	-	-	-	-	-	-
	Exfoliative toxin type D	***etd***	-	-	-	-	-	-	-
	Gamma hemolysin	***hlgA***	-	-	-	-	-	-	-
		hlgB	-	-	-	-	-	-	-
		hlgC	-	-	-	-	-	-	-
	Leukocidin M	***lukF-like***	-	-	-	-	-	-	-
		lukM	-	-	-	-	-	-	-

	Leukotoxin D	***lukD***	-	-	-	-	-	-	-
	Leukotoxin E	***lukE***	-	-	-	-	-	-	-
	Panton-Valentine leukocidin	***lukF-PV***	-	-	-	-	-	-	-
		lukS-PV	-	-	-	-	-	-	-
	Toxic shock syndrome toxin	***tsst***	-	-	-	-	-	-	-
